# Cost-effectiveness of changes in alcohol taxation in Denmark: a modelling study

**DOI:** 10.1186/1478-7547-12-1

**Published:** 2014-01-09

**Authors:** Astrid Ledgaard Holm, Lennert Veerman, Linda Cobiac, Ola Ekholm, Finn Diderichsen

**Affiliations:** 1 Department of Public Health, University of Copenhagen, Copenhagen, Denmark; 2 School of Population Health, The University of Queensland, Brisbane, Australia; 3 National Institute of Public Health, University of Southern Denmark, Copenhagen, Denmark

**Keywords:** Cost-effectiveness, Taxation, Alcohol, Public health, Health effects, Health care costs, Simulation modelling

## Abstract

**Introduction:**

Excessive alcohol consumption is a public health problem in many countries including Denmark, where 6% of the burden of disease is due to alcohol consumption, according to the new estimates from the Global Burden of Disease 2010 study. Pricing policies, including tax increases, have been shown to effectively decrease the level of alcohol consumption.

**Methods:**

We analysed the cost-effectiveness of three different scenarios of changed taxation of alcoholic beverages in Denmark (20% and 100% increase and 10% decrease). The lifetime health effects are estimated as the difference in disability-adjusted life years between a Danish population that continues to drink alcohol at current rates and an identical population that changes their alcohol consumption due to changes in taxation. Calculation of cost offsets related to treatment of alcohol-related diseases and injuries, was based on health care system costs from Danish national registers. Cost-effectiveness was evaluated by calculating cost-effectiveness ratios (CERs) compared to current practice.

**Results:**

The two scenarios of 20% and 100% increased taxation could avert 20,000 DALY and 95,500 DALY respectively, and yield cost savings of -€119 million and -€575 million, over the life time of the Danish population. Both scenarios are thus cost saving. The tax decrease scenario would lead to 10,100 added DALY and an added cost of €60 million. For all three interventions the health effects build up and reach their maximum around 15–20 years after implementation of the tax change.

**Conclusion:**

Our results show that decreased taxation will lead to an increased burden of disease and related increases in health care costs, whereas both a doubling of the current level of alcohol taxation and a scenario where taxation is only increased by 20% can be cost-saving ways to reduce alcohol related morbidity and mortality. Our results support the growing evidence that population strategies are cost-effective and should be considered for policy making and prevention of alcohol abuse.

## Background

Excessive alcohol use is a cause of morbidity and mortality as it increases the risk of many diseases, including several types of cancer and cardiovascular diseases, as well as the risk of (intentional and unintentional) injuries [1-7]. Alcohol consumption is widespread in Denmark. In 2010 the average annual consumption among Danes over the age of 14 was 11.3 litres of pure alcohol [8] and 13% of men and 8% of women exceed the high risk threshold of maximum 14 standard drinks^a^ per week for women and 21 standard drinks per week for men set out by the Danish Health and Medicines Authority [9]. As a consequence, about 6% of the burden of disease in Denmark is due to alcohol consumption, according to the new estimates from the Global Burden of Disease 2010 study [10]. Economically, excess alcohol consumption has been estimated to cost society more than 1% of the gross national product in high- and middle-income countries [11]. A Danish analysis of the socio-economic consequences of alcohol consumption estimated the overall costs of alcohol consumption to be between €160 million using the friction cost method and €1.1 billion using the human capital method in 2005 [12]. Thus, there is a need for interventions to prevent alcohol-related diseases and injuries.

In order to decrease the alcohol related burden of disease, different interventions can be implemented to lower the level of alcohol consumption in entire populations or in specific high risk groups. These include individually focussed programmes, such as education and persuasion or early intervention and treatment services; and legislative policies, which reduce the availability of alcohol, restrict marketing or increase the price of alcoholic beverages [13]. Previous research has found that increased taxation, which raises the price of alcohol, is a very effective way to decrease the burden of disease related to consumption of alcohol [14-18]. Further, economic studies have found that prevention of alcohol related morbidity and mortality through increased taxation is very cost-effective [19-21]. Several legislative preventive alcohol interventions, including increased taxation, were recently highlighted in a policy recommendation for increased public health in Denmark [22]. However, in Denmark decreases in alcohol taxation can be seen as a means to counter cross-border trade in Germany and keep sales within Denmark. A recent example of this was a 15% decrease of the taxation on beer effective from July 1^st^ 2013.

In this paper we estimate the cost-effectiveness of three different alcohol taxation scenarios - two levels of increased taxation and one of decreased taxation - using a dynamic model of the entire Danish population. In the analyses we take a Danish health sector perspective and make use of the unique Danish registers for information on disease incidence, mortality and costs for all individuals in the Danish population.

## Methods

To estimate the cost-effectiveness of changed alcohol taxation, we simulated the impact of changed taxation on population health over the lifetime of the adult Danish population (aged 16 years and older) in 2009. The health effects of taxation are estimated as the difference in disability-adjusted life years (DALYs) [23] between a Danish population that continues to drink alcohol at current rates and an identical population that changes alcohol consumption due to tax increases or decreases. The health-adjusted years of life lived by each of the two populations are calculated by simulating the population in a multi-state life table until everyone has either died or reached 100 years of age. Years of life lived are adjusted at each age for time spent in poor health due to disease or injury.

The model simulated intervention effects on health outcomes associated with alcohol consumption, which included: ischaemic heart disease, ischaemic and hemorrhagic stroke, hypertensive heart disease, pancreatitis, cirrhosis, alcohol dependence, and cancer of the breast (in women), mouth and oropharynx, oesophagus, liver, larynx, colon and rectum [1-6,24]. Further, excess consumption of alcohol increases the risk of a wide range of injury outcomes due to road traffic accidents (RTA) and other accidents (non-RTA) [7,25,26]. Non-RTA injuries associated with death or disability due to alcohol consumption included falls, fires, burns and scalds, drowning, other accidents, suicide and self-inflicted injuries, and homicide and violence [27].

Each of the alcohol-related diseases is modelled by transitions between four states (healthy, diseased, dead from the disease, and dead from all other causes), based on rates of mortality, incidence, case fatality and remission [28]. For injuries, which are acute in nature, changes due to alcohol interventions are modelled through direct changes in incidence of injury-related mortality and disability. Data on incidence and mortality were taken from The Danish National Patient Register [29] and The Danish Register of Causes of Death [30], both of which cover the entire Danish population and can be linked for all individuals. Case fatality (defined as the proportion of prevalent cases that dies in a given year) was calculated using DISMOD II [28].

Average disability associated with each disease (disability weights) was derived from Australian Burden of Disease calculations [31], since such calculations were not available for Denmark. Adjustment for future changes in disease incidence and case fatality was based on trend analysis of Danish mortality and incidence rates by cause^b^.

### Disease outcomes

For all outcomes except alcohol dependence and injuries the intervention effect on disease incidence was modelled by a modified version of the potential impact fraction, where the intervention effect changed the relative risk of disease rather than the population prevalence within alcohol consumption groups (Equation 1) [32,33].

(1) $$\text{PIF}=\frac{\overset{n}{\underset{i=1}{\sum }}p_{i}\;RR_{i}-\overset{n}{\underset{i=1}{\sum }}p_{i}\;\mathrm{RR}{'}_{i}}{\overset{n}{\underset{i=1}{\sum }}p_{i}\;RR_{i}}$$

where:

PIF is the potential impact fraction;

p_*i*_ is the prevalence of alcohol consumption at exposure level *i*;

RR_*i*_ is the relative risk of disease associated with alcohol consumption at exposure level *i*; and

RR'_*i*_ is the relative risk of disease associated with alcohol consumption after an intervention is implemented in the population at exposure level *i*.

Estimates of relative risks were derived from existing meta-analyses (Table 1).

**Table 1:** Baseline relative risks of disease due to alcohol consumption

		**Alcohol intake level**^**a**^				
**Disease**	**Sex**	**Abstinence**	**Low**	**Hazardous**	**Harmful**	**Source**
Ischaemic heart disease (15–34 yr)	Male	0.95 (0.83–1.08)	0.64 (0.17–1.47)	0.56 (0.17–1.19)	1.00	Roerecke and Rehm, [34]
	Female	0.93 (0.89–0.97)	0.34 (0.12–0.67)	0.34 (0.06–0.87)	1.01 (0.05–4.69)	
Ischaemic heart disease (35–64 yr)	Male	0.97 (0.90–1.04)	0.75 (0.38–1.23)	0.70 (0.37–1.10)	1.00	Roerecke and Rehm, [34]
	Female	0.96 (0.94–0.98)	0.53 (0.31–0.77)	0.51 (0.21–0.90)	0.87 (0.20–2.36)	
Ischaemic heart disease (65 + yr)	Male	1.00 (0.96–1.03)	0.99 (0.64–1.39)	0.98 (0.62–1.42)	1.00	Roerecke and Rehm, [34]
	Female	1.00 (0.97–1.03)	0.99 (0.54–1.66)	1.00 (0.46–1.86)	1.02 (0.50–1.75)	
Ischaemic stroke	Male	1.00	0.87 (0.81–0.93)	0.97 (0.90–1.04)	1.24 (1.12–1.37)	Patra et al., [2]
	Female	1.00	0.84 (0.76–0.91)	0.84 (0.74–0.94)	0.98 (0.86–1.12)	
Hemorrhagic stroke	Male	1.00	1.10 (1.06–1.14)	1.27 (1.15–1.40)	1.77 (1.40–2.20)	Patra et al., [2]
	Female	1.00	0.66 (0.52–0.83)	0.76 (0.57–0.99)	1.13 (0.81–1.54)	
Hypertensive heart disease	Male	1.00	1.12 (1.09–1.14)	1.33 (1.25–1.41)	1.95 (1.69–2.24)	Taylor et al., [1]
	Female	1.00	0.80 (0.69–0.92)	1.15 (0.89–1.45)	2.39 (1.61–3.42)	
Pancreatitis	Male	1.00	1.02 (1.02–1.03)	1.16 (1.12–1.20)	2.26 (1.88–2.69)	Irving et al., [3]
	Female	1.00	1.01 (1.01–1.01)	1.05 (1.04–1.07)	1.34 (1.25–1.34)	
Cirrhosis	Male	1.00	1.23 (1.17–1.28)	1.70 (1.51–1.90)	3.49 (2.63–4.53)	Rehm et al., [4]
	Female	1.00	1.82 (1.63–2.04)	2.76 (2.27–3.32)	4.81 (3.55–6.35)	
Breast cancer	Male	–	–	–	–	Corrao et al., [6]
	Female	1.00	1.06 (1.05–1.07)	1.17 (1.14–1.21)	1.47 (1.38–1.57)	
Mouth and oropharynx cancer	Male	1.00	1.37 (1.33–1.41)	2.13 (2.00–2.27)	4.58 (4.13–5-06)	Corrao et al., [6]
	Female	1.00	1.18 (1.16–1.20)	1.59 (1.53–1.66)	2.77 (2.55–2.99)	
Oesophagus cancer	Male	1.00	1.17 (1.16–1.18)	1.51 (1.47–1.54)	2.59 (2.45–2.74)	Corrao et al., [6]
	Female	1.00	1.09 (1.08–1.09)	1.27 (1.26.1.29)	1.78 (1.72–1.84)	
Liver cancer	Male	1.00	1.09 (1.06–1.12)	1-24 (1.15–1-34)	1.59 (1.36–1.85)	Corrao et al., [6]
	Female	1.00	1.05 (1.03–1.06)	1.14 (1.09–1.19)	1.35 (1.22–1.49)	
Larynx cancer	Male	1.00	1.19 (1.17–1.21)	1.55 (1.49–1.62)	2.76 (2.50–3.03)	Corrao et al., [6]
	Female	1.00	1.09 (1.08–1.10)	1.30 (1.26–1.33)	1.85 (1.75–1.97)	
Colon cancer	Male	1.00	1.02 (1.01–1.04)	1.06 (1.02–1.11)	1.15 (1.04–1.27)	Corrao et al., [6]
	Female	1.00	1.01 (1.00–1.02)	1.04 (1.01–1.06)	1.09 (1.03–1.15)	
Rectal cancer	Male	1.00	1.04 (1.03–1.05)	1.12 (1.09–1.15)	1.29 (1.21–1.38)	Corrao et al., [6]
	Female	1.00	1.02 (1.02–1.03)	1.07 (1.05–1.08)	1.17 (1.12–1.21)	

Values are mean relative risk and 95% confidence interval at average alcohol consumption for each consumption category, calculated by Monte Carlo analysis with 3000 iterations.

^a^Alcohol consumption levels: Abstinence (<1.7 g/day), low (1.7-11.9 g/day for women and 1.7-23.9 g/day for men), hazardous (12–23.9 g/day for women and 24–35.9 g/day for men) and harmful (>24 g/day for women and >36 g/day for men).

### Alcohol dependence

For alcohol dependence, which is wholly attributable to excess alcohol consumption, the intervention effect on disease incidence was modelled, by age and sex, as a reduction in the incidence of alcohol dependence that is proportional to the change in consumption at a harmful level of alcohol consumption (Equation 2).

(2) $$j'=j\left(1-\frac{\Delta C_{\text{Harm}}}{C_{\text{Harm}}}\right)$$

where:

*j'* is the incidence of alcohol dependence after an intervention is implemented in the population;

*j* is the current incidence of alcohol dependence, adjusted for level of dependence among harmful drinkers;

Δc_*Harm*_ is the average change in alcohol consumption in the population due to an intervention, in g/day, among those currently drinking at a harmful level;

c_*Harm*_ is the average alcohol consumption, in g/day, among those currently drinking at a harmful level.

### Injuries

The effects of alcohol interventions on injuries were measured by translating a change in alcohol consumption into a change in mortality and morbidity from injuries, by calculating Alcohol-Attributable Fractions (AAF), an adaptation of the potential impact fraction (PIF), as outlined by Taylor et al. [25], and personal communication with Jürgen Rehm (Equation 3).

(3) $$\text{AAF}=\frac{\left(P_{\text{abs}}RR_{\text{abs}}+\overset{n}{\underset{i=1}{\sum }}P_{\text{non}-\text{binge}\left(i\right)}RR_{\text{non}-\text{binge}\left(i\right)}+\overset{n}{\underset{i=1}{\sum }}P_{\text{binge}\left(i\right)}RR_{\text{binge}\left(i\right)}\right)-\left(P_{\text{abs}}RR_{\text{abs}}+\overset{n}{\underset{i=1}{\sum }}P_{\text{non}-\text{binge}\left(i\right)}\mathrm{RR}{'}_{\text{non}-\text{binge}\left(i\right)}+\overset{n}{\underset{i=1}{\sum }}P_{\text{binge}\left(i\right)}\mathrm{RR}{'}_{\text{binge}\left(i\right)}\right)}{\left(P_{\text{abs}}RR_{\text{abs}}+\overset{n}{\underset{i=1}{\sum }}P_{\text{non}-\text{binge}\left(i\right)}RR_{\text{non}-\text{binge}\left(i\right)}+\overset{n}{\underset{i=1}{\sum }}P_{\text{binge}\left(i\right)}RR_{\text{binge}\left(i\right)}\right)}$$

where:

P_abs_ and RR_abs_ is the prevalence and relative risk for current abstainers.

P_binge(i)_ and RR_binge(i)_ is the prevalence and relative risk for current drinkers who engage in binge drinking, for alcohol consumption at exposure level *i* (low, hazardous or harmful). RR for current drinkers who engage in binge drinking is calculated based on average amount of alcohol consumed at each binge episode (this information was not available for Denmark, but based on [35]), time at risk in binge episodes (based on metabolism rates) and average consumption level when not engaging in binge drinking [25].

P_non-binge(i)_ and RR_non-binge(i)_ is the prevalence and relative risk for current drinkers who do not engage in binge drinking, for alcohol consumption at exposure level *i*.

RR'_binge(i)/non-binge(i)_ is the relative risk of injury associated with alcohol consumption at exposure level *i* after an intervention is implemented, for current drinkers who do/do not engage in binge drinking at exposure level *i*. The intervention scenarios analysed are not specifically targeted binge drinking, therefore we do not assume a special effect on binge drinking.

### Intervention scenarios

To evaluate the potential effects of changes in alcohol taxation we analysed three different intervention scenarios and compared these with the current Danish taxation scenario (in the baseline year 2009). Input data for each scenario and model assumptions are shown in Table 2.

**Table 2:** Input data and model assumptions

**Change in taxation**	**20% increase**	**100% increase**	**10% decrease**	**Source**
Intervention effect (mean (SD)) [Distribution]	-1.4% (0.1)	-6.9% (0.7)	0.7% (0.1)	National Danish prevention taskforce [22]. Based on estimates from the ministry of taxation
	[Normal]	[Normal]	[Normal]	
Tax level after tax change (Price per litre pure alcohol)	Beer: €8.2	Beer: €13.6	Beer: €6.1	National Danish prevention Taskforce [22]. Based on estimates from the Ministry of Taxation
	Wine: €8.1	Wine: €13.4	Wine: €6.0	
	Spirits: €24.1	Spirits: €40.2	Spirits: €18.1	
Target population	Current Danish population			-
Proportion of population	100% of non-abstainers			-
Price elasticity	Beer: -0.2, wine: -0.25, spirits: -0.3			National Danish prevention taskforce [22]. Based on estimates from the ministry of taxation
Time horizon	100 years			-
Effect decay rate	2%			Reflecting the rate of inflation; statistics Denmark [36]
Discount rate (costs and effects)	3%			-
Intervention costs [Distribution]	None			National Danish prevention taskforce [22]
	[None]			
Cost offsets [Distribution]	Calculated based on Danish cost data			See ‘Methods’ for details on calculation methods
	[Normal]			
Estimates of Relative risk [Distribution]	See Table 1			-
	[Normal (ln RR)]			

### Current taxation scenario

In Denmark alcohol taxes vary according to beverage type, with heavier taxation on spirits than wine and beer. In 2009 the tax on beer, wine and spirits was €6.8, €6.7 and €20.1 respectively per litre of pure alcohol, or €0.1 per 33 cl bottle or can of beer, €0.6 per 75 cl bottle of wine and €5.6 per 70 cl bottle of spirits. Actual prices are highly dependent on the specific brand, outlet etc., but approximate (low-end) prices for a 33 cl bottle or can of beer, a 75 cl bottle of wine and a 70 cl bottle of spirits are €0.5-€1.2, €5-€13 and €11-€16 respectively.

### 20% increased taxation scenario

In the first scenario we modelled the cost-effectiveness of a 20% increase in alcohol taxation on beer, wine and spirits. This corresponds to a tax increase of €0.02 per 33 cl bottle or can of beer, €0.12 per 75 cl bottle of wine and €1.12 per 70 cl bottle of spirits.

### 100% increased taxation

Here we estimate the health effects and costs of a scenario where the tax levels are doubled compared to the current level of alcohol taxation. This percentage change in taxation would apply to beer, wine and spirits, raising the tax by €0.1 per 33 cl bottle or can of beer, €0.6 per 75 cl bottle of wine and €5.6 per 70 cl bottle of spirits.

### 10% decreased taxation

Finally we estimate the effects of a 10% decrease in alcohol taxation compared to the current level of taxation, decreasing the tax by €0.01 per 33 cl bottle or can of beer, €0.06 per 75 cl bottle of wine and €0.56 per 70 cl bottle of spirits.

In all intervention scenarios it was assumed that the full price change due to changed taxation was passed on from producers to consumers. Taxation is a population wide intervention, assumed to affect the entire adult population of 16 years or older (except abstainers). The effect of taxation on alcohol consumption was measured as relative change in grams of alcohol consumed per day, which was added to the baseline average consumption for each consumption group. For the two tax increase scenarios these estimates of effect were taken from the work done by a National Danish Prevention Taskforce, commissioned to examine and recommend preventive health interventions to be implemented in Denmark [22], and personal communication with Jürgen Rehm. For the tax decrease scenario estimates of effect were based on calculations done by The Danish Ministry of Taxation [37]. Change in alcohol consumption due to changed alcohol taxation was estimated based on price elasticity estimated by The Danish Ministry of Taxation (-0.2 for beer, -0.25 for wine, -0.3 for spirits). In our model we assume a rate of 2% for intervention effect, representing the rate of inflation [36] (Table 2).

### Current alcohol consumption

**Figure 1:**
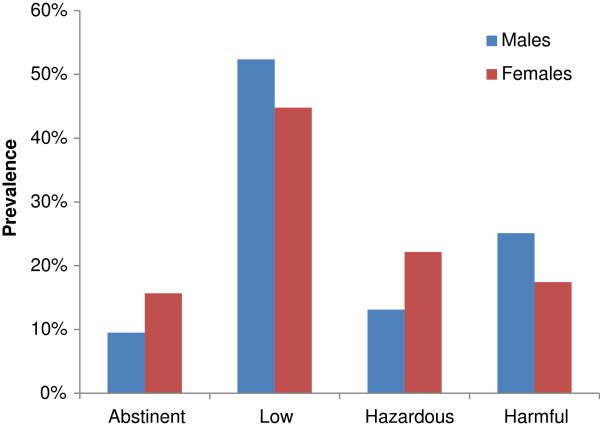
**Distribution of the Danish population on alcohol consumption categories (data from The Danish Health and Morbidity Survey 2010 **[38]**).**

Data collected in The Danish Health and Morbidity Survey 2010 (the national sample in the Danish National Health Survey) were used to determine the current consumption of alcohol [38]. Alcohol consumption was divided into four levels based on the Danish Health and Medicines Authority recommendations for alcohol consumption [39], recalculated into grams per day: Abstinence (<1.7 g/day), low (1.7-11.9 g/day for women and 1.7-23.9 g/day for men), hazardous (12–23.9 g/day for women and 24–35.9 g/day for men) and harmful (>24 g/day for women and >36 g/day for men). Age-specific distributions on consumption levels and average alcohol consumption at each level were calculated. Population distribution on consumption levels are shown in Figure 1. Calculation of injury outcomes (Equation 3) required an estimate of drinking pattern, which was based on frequency of binge drinking (defined as more than five standard drinks in one occasion) from the Danish Health and Morbidity Survey 2010.

Studies have shown that population surveys tend to underestimate alcohol consumption [40,41]. We therefore adjusted for underreporting, using the coverage rate between survey data and adult per capita alcohol consumption from sales statistics (as described by Rehm et al. [42]). To avoid over-adjustment, we assumed that 10% of adult per capita consumption estimated from sales statistics was not consumed (as suggested by Taylor et al. [25]).

### Health effects and net costs

Net costs of each intervention were calculated as the sum of intervention costs and costs of prevented health care utilisation (cost offsets) related to treatment of alcohol-related diseases and injuries. Costs were estimated from a Danish health sector perspective. Intervention costs included government and local government costs associated with deliverance and enforcement of interventions and costs of materials, but excluded costs associated with lost productivity, time costs for patients due to participation in interventions or costs to others (e.g. family). Costs associated with research and development was also excluded.

Cost offsets were evaluated using data on costs of treatment in the health care system, including inpatient and out-patient costs (from the Diagnosis Related Grouping (DRG) Register [43]), data on costs related to consultations with general practitioners and specialists (from the Danish National Health Service Register [44]), data on costs of pharmaceuticals (from the Danish National Prescription Register [45]) and data on municipal health care costs, such as nursing homes, home nursing, rehabilitation etc. Municipal health care costs are not reported consistently across municipalities in Denmark, and we therefore assessed costs in the municipality of Copenhagen^c^ (based on data from the Health and Care Administration of the City of Copenhagen[personal communication], linked to register data on health outcomes via Statistics Denmark). Cost offsets excluded costs to the individual or caregivers, costs due to lost productivity or costs associated with alcohol-related crime and violence. In the absence of a Danish cost of illness study, cost offsets were quantified for each of the included health outcomes by comparing health care costs for people with the disease and health care costs for people without the disease. Multiple regression analyses were used, mutually controlling for all included health outcomes and for other diseases not associated with alcohol consumption. Average health care costs due to diseases not associated with alcohol consumption were included in the analysis in order to account for costs in added years of life. Costs were derived by sex and age (<49, 50–69, 70+ years), based on rates of disease in 2009.

### Cost-effectiveness analyses

Costs and effects of the three intervention scenarios were plotted on the cost-effectiveness plane to illustrate cost-effectiveness. Cost-effectiveness ratios (CERs) were calculated as the ratio of means [46] and compared to the current taxation scenario. All costs and effects were evaluated for the Danish population with a lifetime perspective, and a discount rate of 3% per annum was used for both costs and health outcomes. Probabilities of the interventions being cost-effective or cost saving were modelled using cost-effectiveness acceptability curves. We used WHO’s thresholds of less than GDP per capita GDP per capita for highly cost-effective and between one and three times GDP per capita for cost-effective, since there is no agreed threshold of cost-effectiveness in Denmark (GDP Denmark, 2009: €39,900 [47]).

The statistical software SAS (version 9.2) was used for analyses of epidemiological data inputs and cost data. The cost-effectiveness analyses were performed in Excel (Microsoft Office 2007), applying the add-in programme Ersatz (version 1.31, Epigear 2012) for uncertainty analyses.

### Uncertainty and sensitivity analyses

We used Monte Carlo simulation for uncertainty analyses in the cost-effectiveness model. We assessed the possible effects of uncertainty in estimates of relative risk, effects, costs and coverage rates of the intervention and cost offsets.

In sensitivity analyses we tested the effect of three main assumptions made in the modelling. The price elasticity estimates used in the main analysis are calculated by The Danish Ministry of Taxation [22]. To test the effects of this choice of price elasticity we did a sensitivity analysis where estimates of price elasticity are based on a meta analysis by Gallet [48]. The price elasticities used in this analysis are -0.35, -0.7 and -0.7 for beer, wine and spirits respectively.

In our main analysis we assumed changes in alcohol taxation to be cost-neutral in terms of intervention costs [22]. In a sensitivity analysis we tested the implications of this assumption, by calculating the cost-effectiveness of the three taxation interventions assuming that changed taxation would involve a yearly cost of €375,000, plus a cost of €270,000 the first year. This is the estimated cost of law enforcement activities plus start up expenses and added information the first year, applied to other legislative interventions such as changes in hours of retail sale or legal drinking age [22].

The proportion of a tax increase that is passed on to consumers may be less than, equal to, or greater than the full change in taxation. In the main analysis we assumed a tax pass-through rate of one, indicating that the full tax increase is passed on to consumers. In the third sensitivity analysis we tested the implications of this tax pass-through rate by applying two alternative rates of tax pass-through based on the studies by Young and Bielinska–Kwapisz (average pass-through rate of 1.66) and Kenkel (average pass-through rate of 2.57) [49,50].

## Results

If the tax on alcohol was increased by 20%, 20,000 DALY could be averted. The effect of a doubling of the alcohol tax is substantially larger, with 96,000 DALY averted. A 10% tax reduction on the other hand would result in 10,000 additional DALY. The cost offsets for the tax increase scenarios were €119 million and €575 million respectively, and for the tax decrease scenario a negative cost offset of -€60 million. The cost-effectiveness ratios are dominant for both scenarios of increased taxation, indicating that the interventions are cost saving and health promoting, whereas the cost-effectiveness ratio for the decreased taxation scenario is dominated. Table 3 shows differences in health gains, costs and cost-effectiveness ratios for the three scenarios.

**Table 3:** Cost-effectiveness of alcohol taxation interventions for the Danish population aged 16+ (population in 2009: 4.5 million)

**Intervention**	**DALYs averted**^**a**^			**Cost offsets (€ million)**			**ICER**^**b **^**(€/DALY)**		
	**Mean**	**CI95% low**	**CI95% high**	**Mean**	**CI95% low**	**CI95% high**	**Mean**^**c**^	**CI95% low**	**CI95% high**
20% increase	19,986	16,113	23,929	-118.9	-148.7	-90.6	Dominant	Dominant	Dominant
100% increase	95,536	77,413	114,020	-575.2	-717.7	-439.7	Dominant	Dominant	Dominant
10% decrease	-10,108	-12,093	-8,067	60.0	46.2	75.1	Dominated	Dominated	Dominated

^a^DALY = disability-adjusted life year. ^b^ICER = incremental cost-effectiveness ratio. ^c^Calculated as ‘ratio of means’ [46].

**Figure 2:**
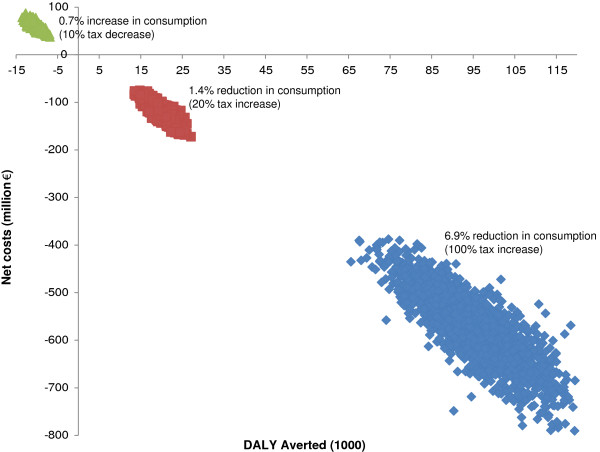
Cost-effectiveness of alcohol taxation scenarios.

The two scenarios with increased taxation are positioned in the south-east quadrant of the cost-effectiveness plane, indicating that these interventions are cost saving (Figure 2). Opposite, the taxation decrease scenario is positioned in the north-west quadrant, indicating that this level of taxation is less effective and more costly than current practice.

**Figure 3:**
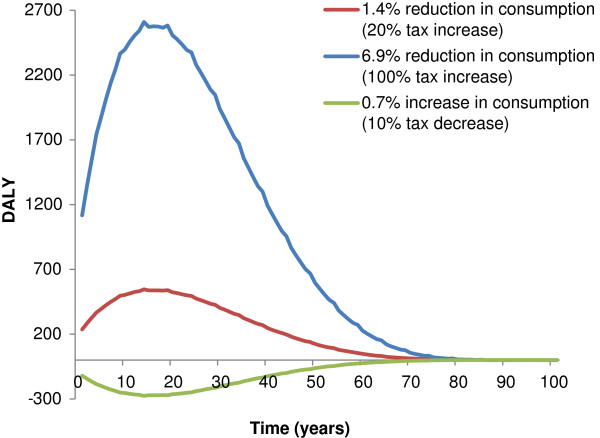
Health effects of alcohol taxation over the modelled timeframe for the Danish population aged 16+ in 2009 (population 4,5 million).

The health effects of changed alcohol tax over the modelled timeframe are illustrated in Figure 3. For the two scenarios with increased taxation, the lower consumption of alcohol reduces the incidence of alcohol-related diseases and the number of disability adjusted life years experienced by the population compared to current practice. For the scenario with decreased taxation the opposite effect can be observed, with an increase in disability adjusted life years. The health effects of taxation build up and are largest around 15–20 years after the change in taxation. As the simulated cohorts gradually age and dwindle in numbers, the health effects diminish.

**Figure 4:**
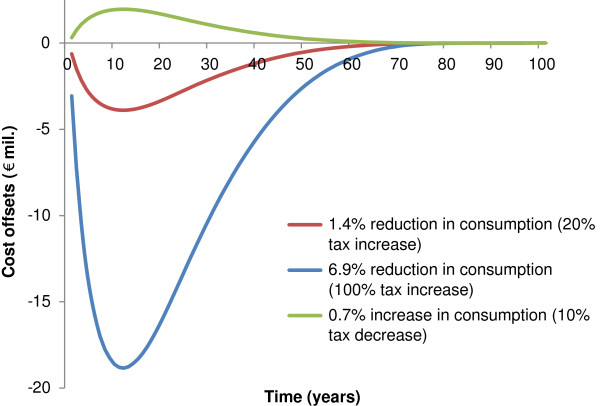
Cost offsets due to alcohol taxation over the modelled timeframe for the Danish population aged 16+ in 2009 (population 4,5 million).

For the decreased taxation scenarios the reduced incidence of alcohol related diseases leads to lower health care costs associated with these diseases. As illustrated in Figure 4, these savings are reduced over time due to increased costs associated with people living longer, but also due to discounting of future costs (Table 2). For the tax increase scenario we see the opposite, with increased health care costs, which are also reduced over time.

### Sensitivity analyses

Applying alternative, and higher, estimates of price elasticity resulted in larger health effects of all three scenarios. In this analysis the 20% tax increase could avert 44,000 DALY in a 100 year modelling frame and 200,000 DALY could be averted with a doubling of the current level of taxation. With the alternative estimates of price elasticity, a 10% reduction of alcohol taxation could increase the burden of disease by 23,000 DALY with (Table 4).

**Table 4:** Sensitivity analyses: cost-effectiveness of alcohol taxation under alternative assumptions (for the Danish population aged 16+ in 2009, population: 4.5 million)

**Intervention**		**DALYs averted**^**a**^			**Cost offsets (€ million)**			**ICER**^**b **^**(€/DALY)**		
		**Mean**	**CI95% low**	**CI95% high**	**Mean**	**CI95% low**	**CI95% high**	**Mean**^**c**^	**CI95% low**	**CI95% high**
Price elasticity^d^	20% increase	43,873	35,130	52,556	-263	-332	-201	Dominant	Dominant	Dominant
	100% increase	204,291	167,407	243,095	-1,246	-1,584	-973	Dominant	Dominant	Dominant
	10% decrease	-23,209	-27,760	-18,645	137	107	173	Dominated	Dominated	Dominated
Intervention cost^e^	20% increase	19,995	16,080	24,007	-119	-148	-92	Dominant	Dominant	Dominant
	100% increase	95,639	76,949	113,174	-575	-718	-440	Dominant	Dominant	Dominant
	10% decrease	-10,085	-12,107	-8,077	60	46	75	Dominated	Dominated	Dominated
Taxation pass-through rate of 1.66^f^	20% increase	33,282	26,760	40,024	-197	-247	-152	Dominant	Dominant	Dominant
	100% increase	157,148	129,415	186,267	-944	-1,182	-733	Dominant	Dominant	Dominant
	10% decrease	-17,010	-20,368	-13,793	100	77	126	Dominated	Dominated	Dominated
Taxation pass-through rate of 2.57^f^	20% increase	50,580	40,628	60,523	-301	-379	-229	Dominant	Dominant	Dominant
	100% increase	239,017	191,170	291,742	-1,518	-2,194	-1,104	Dominant	Dominant	Dominant
	10% decrease	-26,348	-31,770	-21,220	154	119	194	Dominated	Dominated	Dominated

^a^DALY = disability-adjusted life year. ^b^ICER = incremental cost-effectiveness ratio. ^c^Calculated as ‘ratio of means’ [46]. ^d^In the first sensitivity analysis the effects of higher price elasticities of -0.35, -0.7 and -0.7 respectively for beer, wine and spirits are tested. ^e^In the second sensitivity analysis the effects of assuming the same intervention cost for taxation as for other legislative interventions (yearly cost of €375,000 plus €270,000 the first year) are tested. ^f^In the third sensitivity analysis the effects of the assumed taxation pass-through rate of 1 is tested by applying two alternative pass-through rates from Young and Bielinska–Kwapisz (a rate of 1.66) and Kenkel (a rate of 2.57) [49,50].

The results of assuming an intervention cost for the taxation interventions similar to the estimated cost of other legislative alcohol interventions in Denmark are also shown in Table 4. It can be seen that for all three intervention scenarios, cost-effectiveness is only affected marginally by the change in intervention cost.

The two alternative estimates of pass-through rate of taxation increase the absolute effect off the interventions considerably (Table 4). The pass-through rate found by Kenkel was the higher of the two and thus resulted in the largest effect on both costs and effects.

## Discussion

We modelled the potential health effects and associated costs of two different scenarios of increased alcohol taxation and one scenario of decreased alcohol taxation in Denmark. Our model was based on epidemiological evidence and Danish register data of high quality. However, the intervention scenarios analysed are modelled *scenarios*; not observed policy changes. We found that both scenarios with increased tax were cost-saving. This is in line with previous research, which has found increased taxation to be a cost-effective or cost-saving way of preventing alcohol related diseases and injuries [19-21]. From a health sector perspective we found that the scenario of decreased taxation is a less effective and more costly alternative to current practice.

Due to price differences compared to our neighbouring countries, cross-border trade is relatively common in Denmark, with Danes buying alcohol and other goods in Germany and Swedes purchasing goods in Denmark. A way to counter the cross-border trade with Germany and increase sales in Denmark could be to decrease taxation of alcohol. Based on revenue calculations it has been argued that tax decreases could be cost saving, however, these calculations most often do not include health care costs or other broader cost to society [51].

A few studies have examined the effects of a decrease in the taxation on spirits by 45% in 2003 on alcohol consumption. Two studies reach divergent results: Based on panel data, both studies found that consumption of spirits decreased by 7-8% in the year following the decrease in taxation, however, based on repeated cross sectional data a 6-11% increase in total consumption was found [52,53]. In our study we assumed that a 10% tax decrease would result in a 0.7% increase in consumption, applied to cross sectional consumption data. This estimate of change is cautious compared to the estimates found for cross sectional data in the two studies. Unfortunately our estimates of cost-effectiveness cannot be compared to the consequences of the decrease in spirits tax, since the long term health effects have not yet been studied. Unlike the scenarios analysed in our study, this policy affected the taxation of spirits only, and substitution between types of alcohol might have occurred.

We only modelled the health effects in a population aged 16 years and older, but chances in alcohol taxation might also affect the younger population. Another study of the effects of the tax decrease on spirits in Denmark in 2003 found that the number of hospitalizations due to acute alcohol intoxication increased by 26% among people aged 15 years or younger [54].

Other studies have modelled the cost-effectiveness of potential changes in alcohol taxation, most with a focus on tax increases. Due to differences in modelled scenarios, modelling approaches and characteristics of the populations modelled, it is difficult to make direct comparisons between our results and the findings from other studies. However, subject to these differences, our estimates of potential health effects are lower than found by Chisholm et al., Purshouse et al. and van den Berg et al. [14,19,21], but higher than found by Cobiac et al. and Lhachimi et al. [18,20], as illustrated in Table 5. Generally we find larger potential cost offsets compared to these other studies.

**Table 5:** Overview of findings in other studies of increased taxation

**Study**	**Taxation change**	**Study population and time frame**	**Effect**	**Intervention cost**	**Cost offsets**	**CER**
Cobiac et al. [20]	Volumetric tax	Australia 100 years	11,000 DALY	AU$0.58 million	-AU$57	Dominant
Chisholm et al. [21]	25% increase	Western Europe 10 years (costs and effects p.a.)	1,500 DALY per 1 million population	I$0.45 million per 1 million population	-	289 I$/DALY
Van den Berg et al. [19]	34% increase for beer	Netherlands	13,000 QALY	None	€65 million	5100 €/QALY
	225-300% increase^*^	100 years	625,000 QALY		€3300 million	5300 €/QALY
Lhachimi et al. [18]	20% tax increase	European Union	19,100 deaths prevented (400 in Denmark)	-^**^	-^**^	-^**^
	80% tax increase	10 years	107,800 deaths prevented (2300 in Denmark)			
Purshouse et al. [14]	10% general price increase	England 10 years	55,000 QALY	-^**^	-^**^	-^**^

CER = Cost-effectiveness ratio; AU$ = Australian dollar; p.a. = per annum; I$ = International dollar.

^*^ Differentiated increase for beverage types.

^**^ No cost calculations included in study.

Some of these differences in estimates of health effects might be due to differences in alcohol consumption and baseline epidemiology and demography of the populations studied. Denmark has a larger baseline burden of disease from alcohol-related diseases than many otherwise comparable countries [10]. Thus there is a large potential for health prevention through decreased alcohol consumption. However, compared to some studies we find smaller health effects. The estimation of potential health effects are influenced by a range of factors in the modelling, including estimates of price elasticity. The price elasticity estimates used in our study are calculated by The Danish Ministry of Taxation [22]. These estimates are lower than the estimates used in other studies of effect and cost-effectiveness of alcohol taxation [14,18,19,21]. We used these estimates, since they are based on a Danish context. Further, since the level of alcohol taxation is already higher in Denmark compared to many other European countries [18], a tax increase in Denmark could have lower effects, due to increased incentives for cross-border trade. This effect has been taken into account in the lower estimates of price elasticity. In our sensitivity analyses we tested the implications of applying higher price elasticities. The results of this analysis are closer to the effects found in other studies which used higher price elasticity estimates [18,19].

Variations in included and excluded health care costs affect intervention cost-effectiveness, and can explain some of the differences between our estimates of cost-effectiveness and those found in other studies. Further, in our study the tax interventions are assumed to be cost-neutral in terms of intervention costs (Table 2). Our estimates of intervention costs are based on the work done a National Danish Prevention Taskforce, appointed to examine and recommend preventive health interventions to be implemented in Denmark [22]. For increased taxation The Danish Ministry of Taxation estimated that current costs would not change with an increased taxation level. This is not in accordance with WHO’s generalised cost-effectiveness approach where interventions are compared to a null scenario [55]. The results of our sensitivity analysis show that even if taxation was assumed to have costs comparable to those of other legislative interventions, the two scenarios with decreased taxation would still be cost-saving.

We did not include changes in Government revenue in our cost estimates. From a societal perspective it has been argued, that tax revenues should not be included in cost-effectiveness analyses, since they are transfer payments [19,21]. Further, comparable estimates of revenue change were not available for all three taxation scenarios.

In the analysis of health effects over time, we found that the largest health effects were 15–20 years of intervention. This is about 10 years earlier than found by van den Berg and colleagues [19]. This difference is partly attributable to the effect of discounting, where we in our study use a 3% discount rate for effects, compared to the discount rate of 1.5% applied by van den Berg et al., but also attributable differences in the baseline population.

Based on the calculations performed by the Danish Ministry of Taxation, we assumed that increased tax on alcohol will translate fully into increased prices of alcoholic beverages. Only few studies have examined supply-side responses to alcohol pricing policies, which could include market restructuring or increased discounts [56,57]. Kenkel [49] and Young and Bielinska–Kwapisz [50] have analysed alcohol tax pass-through rates in USA, and both found that prices increased by more than the actual tax increase [49,50]. Kenkel found that sales outlets with a higher baseline price passed less of the tax increase on to consumers [49]. This supports the rather cautious pass-on rate of one assumed in our study, since baseline prices are rather high in Denmark. In the sensitivity analyses we applied the higher pass-through rates found by Kenkel and Young and Bielinska–Kwapisz, which enhanced the impact of the analysed tax changes considerably. The assumption regarding taxation pass-through rate thus have implications for our results, and the effect of alcohol taxation on alcohol consumption might therefore be greater than found in our study.

For information on morbidity, mortality and costs we used linked data from national Danish registers. This allowed us to base our analysis on information for the entire Danish population. Alcohol consumption data is based on data from a representative national Danish health survey [38]. We adjusted these data for underreporting, using the coverage rate between survey data and sales statistics. This approach presumes that under-coverage by surveys is evenly distributed in the population, which might not be the case. However, no clear evidence exists on differential underreporting of alcohol consumption by different survey subpopulations [42]. In our analyses we included average daily alcohol consumption and –for the injury calculations only– an estimate of binge drinking. Due to limitations in the available data, we were not able to properly include effects of drinking patterns. Differences have been found in the effect of alcohol intake among regular drinkers and irregular drinkers [58], and this aspect should thus be investigated further in future studies.

In our modelling approach we did not include time lag effects of the temporal relationship between alcohol consumption and incidence of health outcomes. Only few studies have examined this aspect, but a recent review propose that there are immediate effects of changes in alcohol consumption on both mortality and morbidity of many health outcomes, except cancers, and that full effects are obtained after 10 to 20 years [59]. This is in line with our results, where we found that the health effects built up and were largest around 15–20 years after a change in alcohol taxation (Figure 3).

Danish population registers, covering all individuals in the Danish population, were used for information on disease incidence, mortality and costs. In The Danish National Patient Register diseases are registered according to ICD-10 codes, whereas registration of injuries is based on the Nordic Classification of External Causes to Injuries [43]. A weakness in this approach is, that long term disability due to injuries are not linked to injury incidence, and we were thus unable to include these in our cost estimates. A Danish study has found that, depending on diagnosis, the use of health care services was higher among people with serious injuries up to nine years after the injury [60]. This indicates that our cost estimates for injuries might be underestimated.

The disability adjusted life year (DALY) was used as the measure of effect, a measure which has been widely discussed [61,62]. We did not use age weighting, but both effects and costs were discounted by 3%. As argued by Chisholm et al. [63], the main limiting factor related to the use of DALY in cost-effectiveness studies is the inability to include non-health effects or effects on others than the person at risk. An exception being victims of alcohol related accidents and crimes, but the societal potential of the interventions could still be larger than our estimates.

Public policy making should to be informed by evidence; however when evidence from evaluations of previous interventions is not available, results from modelling studies can help guide discussion and decision making by presenting possible outcomes and effects, and the probability and consequences of these. In Denmark there is little history of modelling studies within public health and our study adds new perspectives to discussions regarding the possible effects of changes in alcohol taxation, a frequently debated topic.

## Conclusion

Alcohol is one of the leading risk factors in many industrialized countries, and in Denmark 6% of the total burden of disease is attributable to alcohol consumption. We found that, from a health sector perspective, decreased alcohol taxation, as has been suggested in Denmark, will raise both the burden of disease and health care costs. On the other hand, our study shows that increased taxation of alcohol can be a cost-saving way to reduce alcohol related morbidity and mortality. This applies to both a scenario where the current level of taxation is doubled, but also to a less radical scenario where alcohol taxation is increased by 20%.

## Endnotes

^a^ A Danish standard drink is equivalent to 12 grams pure alcohol [39].

^b^ Mortality trends are based on mortality between 1977 and 2009 for cardiovascular diseases and breast cancer, and between 1994 and 2009 for other cancers and cirrhosis, due to lack of available comparable data [64]. Incidence trends are based on data from 1977 to 2009, but only available for cancers [64]. For pancreatitis and alcohol dependence we assumed stable rates over time.

^c^ The Municipality of Copenhagen, the capital of Denmark, is the largest municipality in the country, with 10% of the Danish population.

## Competing interests

The authors declare that we have no competing interests.

## Authors’ contributions

ALH was the main contributor to data analysis and writing of the paper. LV and LC contributed significantly to the design of the study, data analysis and writing of the paper. OE supplied parts of the data material and contributed with revisions of manuscript drafts. FD contributed to the design of the study and drafting of the article. All authors approved the final version of the manuscript.
